# Burnout and associative emotional status and coping style of healthcare workers in COVID-19 epidemic control: A cross-sectional study

**DOI:** 10.3389/fpubh.2023.1079383

**Published:** 2023-03-09

**Authors:** Cece Yang, Xunqiang Wang, Xing Zhang, Wenping Liu, Chengmin Wang

**Affiliations:** Department of Mental Health, Longgang Center for Chronic Disease Control, Shenzhen, China

**Keywords:** burnout, coping style, anxiety, depression, insomnia, self-efficacy

## Abstract

**Objective:**

The aim of this study was to evaluate the prevalence of burnout, clinical anxiety, depression, and insomnia and to estimate the associations of adverse emotional status, coping style, and level of self-efficacy with burnout of healthcare workers in the Shenzhen Longgang District Frontline District Headquarters of COVID-19 epidemic control, China.

**Methods:**

In this cross-sectional study, 173 staff completed the anonymous questionnaires of the Maslach Burnout Inventory, Patient Health Questionnaire-9 (PHQ-9), Generalized Anxiety Disorder 7-item Scale (GAD-7), Insomnia Severity Index (ISI), General Self-efficacy Scale, and Simplified Coping Style Questionnaire electronically (https://www.wjx.cn/) in June 2022. Hierarchical logistic regression was used to explore the associated factors of burnout in this study.

**Results:**

The prevalence of burnout in our participants (defined as high emotional exhaustion or high depersonalization) was 47.40%, and reduced personal accomplishment was 92.49%. The prevalence of clinically significant depression (the cutoff score of ≥15), anxiety (the cutoff score of ≥10), and insomnia (the cutoff score of ≥15) was 11.56, 19.08, and 19.08%, respectively. There was a degree of overlap between burnout and other measures of adverse mental status, most notably for anxiety (odds ratio, 27.049; 95% CI, 6.125–117.732; *p* < 0.001). Hierarchical logistic regression demonstrated that burnout was strongly associated with anxiety (OR = 23.889; 95% CI, 5.216–109.414; *p* < 0.001) and negative coping style (OR = 1.869; 95% CI, 1.278–2.921; *p* < 0.01) independently.

**Conclusion:**

Medical staff involved in COVID-19 epidemic control in the post-epidemic era were at high risk of burnout, and most of them were in low personal accomplishment. Reducing anxiety and improving coping style by medical management institutions from the system level may be effective in alleviating burnout in healthcare workers.

## Introduction

Viral infections are related to mental health. In the context of the coronavirus disease 2019 (COVID-19) pandemic, people may experience great changes in fear, stress, and daily lives. Depression, anxiety, and insomnia are very common mental health problems during the COVID-19 pandemic (1, 2). Some surveys showed a high prevalence of burnout (3, 4), depression (5, 6), anxiety (5, 6), and insomnia (6–9) in health professionals in the past. In 2020, the prevalence of major depression and anxiety increased by more than 20%, respectively, worldwide and significantly in countries seriously affected by COVID-19. With the continued spread of COVID-19, researchers predicted that the incidence rate of depression and anxiety may increase again (10).

Burnout is defined as an excessive reaction to stress caused by one's environment that may be characterized by feelings of emotional and physical exhaustion, coupled with a sense of frustration and failure. Before the outbreak of the COVID-19 epidemic, medical staff faced a general problem of burnout, which became more prominent during the epidemic (11). More than half of primary care practitioners in China during the COVID-19 epidemic control reported fatigue (12). Socio-demographic factors could be related to burnout (13); in addition, negative emotional states such as depression and anxiety could affect burnout (14), and sleep quality (15), different coping styles (16), and levels of self-efficacy (17, 18) might be protective or risk factors of burnout. There can be complex relationships among these variables. There is a need for more evidence as to which factors are protective, as well as which are at risk of burnout independently. However, less information is available on the association between burnout and mental status, coping style, and self-efficacy in health professionals, especially those who had been involved in COVID-19 prevention and control for a long time in Shenzhen, China.

Healthcare wokers in the Longgang District Frontline Headquarters of COVID-19 epidemic control came from hospitals and public health institutions in Shenzhen, worked on call and in a relatively isolated and closed centralized place after the outbreak of the COVID-19 pandemic. Over the last 2.5 years, medical staff needed to be ready at any time if the epidemic occurred again. The working hours were longer than before (12), the working conditions were more severe (12), and these staff continued to be in a state of high stress and uncertainty. A previous study in Hong Kong and Canada showed that the SARS pandemic outbreak changed primary care practitioners' work environments and lifestyles (19). These medical staff could be at high risk of burnout and negative emotional and insomnia distress. What was the prevalence of their emotional and sleep status? How about the risk of burnout? Do their emotional status, coping style, and self-efficacy possibly affect the incidence of burnout? As the COVID-19 epidemic lasts longer, our bodies and minds can adjust and adapt. Whether the prevalence of mental problems and burnout will increase or not during the late stage of the COVID-19 pandemic needs to be explored in the study.

In this study, we surveyed burnout, mental status, coping style, and levels of self-efficacy by using the Maslach Burnout Inventory-Human Services Survey (MBI-HSS), Patient Health Questionnaire-9 (PHQ-9), Generalized Anxiety Disorder 7-item Scale (GAD-7), Insomnia Severity Index (ISI), General Self-efficacy Scale (GSES), and Simplified Coping Style Questionnaire (SCSQ) among healthcare workers in the Shenzhen Longgang District Frontline Headquarters of COVID-19 epidemic control, China. These health workers worked in a relatively isolated place, which was convenient for sampling and could be representative of the medical staff involved in COVID-19 prevention and control in Longgang District, Shenzhen.

The main aim of this study was to explore the independent relative impact of emotional status, coping style, sleep quality, and self-efficacy on the outcome of burnout, as well as examine the degree to burnout was related to the job nature of healthcare workers and other relevant socio-demographic and occupational factors. A secondary aim was to explore the rates of mental problems and burnout among healthcare workers in China more than 2 years after the outbreak of COVID-19 and establish the degree of overlap between burnout status and anxiety, depression, and insomnia. It was hypothesized that high levels of burnout and anxiety, depression, and insomnia were reported in participants, and negative coping, low levels of self-efficacy, and adverse emotional status would be associated with high rates of burnout.

## Materials and methods

The design was a cross-sectional anonymous survey in the in the Shenzhen Longgang District Frontline Headquarters of COVID-19 epidemic control in June 2022. We released research-related notices in advance in the WeChat work group, and then participants were sent the anonymous questionnaires electronically (https://www.wjx.cn/), which they completed and returned after informed consent. Staff with a history of anxiety disorder and major depressive disorder were excluded from this survey.

### Measures

We collected the socio-demographic characteristics of participants including age, gender, education, marriage, family income, professional position, years since qualified, work nature and length of staying in the Shenzhen Longgang District Frontline Headquarters of COVID-19 epidemic control, physical condition, smoking, and drinking.

#### Patient Health Questionnaire-9

The PHQ-9 contains nine items which are scored on a four-point Likert scale from 0 indicating “not at all” to 3 indicating “nearly every day” and then summed (20). This scale was developed based on DSM-IV criteria for diagnosing major depressive disorders and was used to assess the frequency of depressive symptoms in the past 2 weeks. The range of scores was from 0 to 27, with higher scores representative of worse depression. A cutoff score of ≥15 has been recommended for its good internal consistency and reliability. Depression was defined as a total score of more than or equal to 15 on PHQ-9. Overall Cronbach's alpha of the Chinese version of the PHQ-9 in the general population was 0.86 (21). The reliability in this sample was good (α = 0.906).

#### Generalized Anxiety Disorder 7-item Scale

The GAD-7 contains seven items which are scored on a four-point Likert scale from 0 indicating “not at all” to 3 indicating “nearly every day” and then summed (22). This scale is a self-report questionnaire and assesses symptoms of anxiety over the last 2 weeks. All scores were 0–21, with higher scores indicating worse anxiety. A cutoff score of ≥10 on GAD-7 is used to define anxiety in this study. The reliability in the current sample was good (α = 0.938).

#### Insomnia Severity Index

The ISI is a self-report questionnaire (23), which was a four-point Likert scale, with responses weighted 0–3 for frequency. The score ranged from 0 to 28, with higher scores indicative of severe insomnia. A cutoff score of ≥15 means meeting the diagnostic criteria of clinical insomnia and was used in this study. The reliability and validity of the Chinese Translation of Insomnia Severity Index (C-ISI) are good.

#### Maslach Burnout Inventory

The MBI (24) as the gold standard for evaluating burnout syndrome severity includes 22 items which are scored on a seven-point scale from 0 indicating “never” to 6 indicating “every day”. It is a self-report inventory divided into three subscale dimensions, namely emotional exhaustion (EE), depersonalization (DP), and low personal accomplishment (PA). Greater than 26 for EE, >9 for DP, or <33 for reduced PA means high risk on each dimension. “Burnout” is diagnosed if one has high-risk levels of EE (≥27) or DP (≥10) and given the lack of evidence for PA as a predictor.

#### General Self-efficacy Scale

The GSES (25) contains 10 items which are scored on a four-point Likert scale from 1 to 4. A total score is the average score of ten items, with a higher score indicative of better self-efficacy.

#### Simplified Coping Style and Questionnaire

The SCSQ (26) consists of two dimensions: positive coping and negative coping, and contains 20 items which are scored on a four-point Likert scale from 0 indicating “not adopted” to 3 indicating “always adopted”. This self-report scale is scored by the average score of the positive coping dimension and negative coping dimension separately. The positive coping dimension consists of items 1–12, which mainly reflect the characteristics of positive response, such as “try to see the good side of things” and “seek hobbies and actively participate in sports activities”. The dimension of negative coping consists of items 13–20, which mainly reflect the characteristics of negative coping, such as “relieving worries by smoking and drinking” and “thinking that time will change the status quo, the only thing to do is to wait”. The tendency of coping refers to the standard score of positive coping minus the standard score of negative coping.

## Statistics

Statistical analysis was performed by using the Statistical Package for the Social Sciences, Release 25.0 (SPSS; IBM Corp, Armonk, NY). Descriptive data were shown in the form of the mean (SD) and n (%). Socio-demographic characteristics, the prevalence rate of depression, anxiety, insomnia, burnout, coping style, and level of self-efficacy were described. Only variables showing significant association (i.e., *p* < 0.05) in univariate analyses (chi-square and independent group *t*-tests) were then entered into a hierarchical logistic regression model to determine their independent associations with burnout. A *p*-value of < 0.05 was considered statistically significant.

### Ethics statement

This current study was submitted to and approved by the Control and Prevention Command Office of the COVID-19 pandemic in Longgang District, Shenzhen city, Guangdong Province, and the Longgang Center for Chronic Disease Control of Shenzhen and adhered to the Declaration of Helsinki. All participants provided informed consent before completing the online survey, by reading the instructions along with this study's purpose and significance of the survey. They were guaranteed confidentiality and were asked to choose “yes” or “no” for participating in the survey. If they chose “no”, they would not have to continue the study. Otherwise, the survey would go on. All participants in this study were above the age of 18.

## Results

### Socio-demographic data

Their socio-demographic characteristics of the 173 participants are presented in Table 1. Of the 173 participants in the present study, 99 (57.23%) were women. The mean age was 35.44 ± 7.69 years with a range from 21 to 57 years. 67.05% (*n* = 116) of participants were married. Of these health professionals, 33.53% (*n* = 58) were medical care personnel, and 59.54% (*n* = 103) were public health personnel. More than half of them (53.76%) worked for more than 10 years since they qualified. Approximately, 31.79% (*n* = 55) worked in the epidemiological survey, and 58.96% (*n* = 102) had worked for more than 1 year in the control of COVID-19.

**Table 1:** Characteristics of participants of health professionals (*N* = 173).

**Variable**		***n* (%)**	**%**
Gender	Male	74	42.78
	Female	99	57.23
Age group (year)	< 30	42	24.28
	30–39	79	45.66
	≥40	52	30.06
Educational level	High school and below	5	2.89
	College	143	82.66
	Postgraduate degree	25	14.45
Family income	Low	43	24.28
	Medium	116	67.05
	High	14	8.09
Years since qualified	< 5	47	27.17
	5–10	33	19.08
	>10	93	53.76
Physical condition	Well	79	45.66
	General	84	48.56
	illness	10	5.78
Smoking	Yes	18	10.40
Drinking	Yes	40	23.12

### The prevalence and the degree of overlap of anxiety, depression, insomnia, and burnout

The average score in PHQ-9, GAD-7, and ISI was 7.55 ± 5.38, 5.71 ± 4.84, and 9.29 ± 6.20, respectively. Approximately 11.56% (*n* = 20) of individuals reported clinical depression, 19.08% (*n* = 33) participants showed clinical anxiety, and 19.08% (*n* = 33) scored in clinical insomnia. The proportion of the participants scoring in the high risk for each of the three MBI dimensions measured was as follows: EE 34.68% (*n* = 60), DP 41.62% (*n* = 72), and reduced PA 92.49% (*n* = 160). The proportion meeting the criterion for burnout in this study (EE or DP) was 47.40% (*n* = 82). The average score of the positive coping in the SCSQ was 1.59 ± 0.59. The average score of the negative coping in the SCSQ was 1.22 ± 0.54. The tendency of coping was 0.37 ± 0.64. The average score of the GSES was 2.36 ± 0.61.

**Figure 1:**
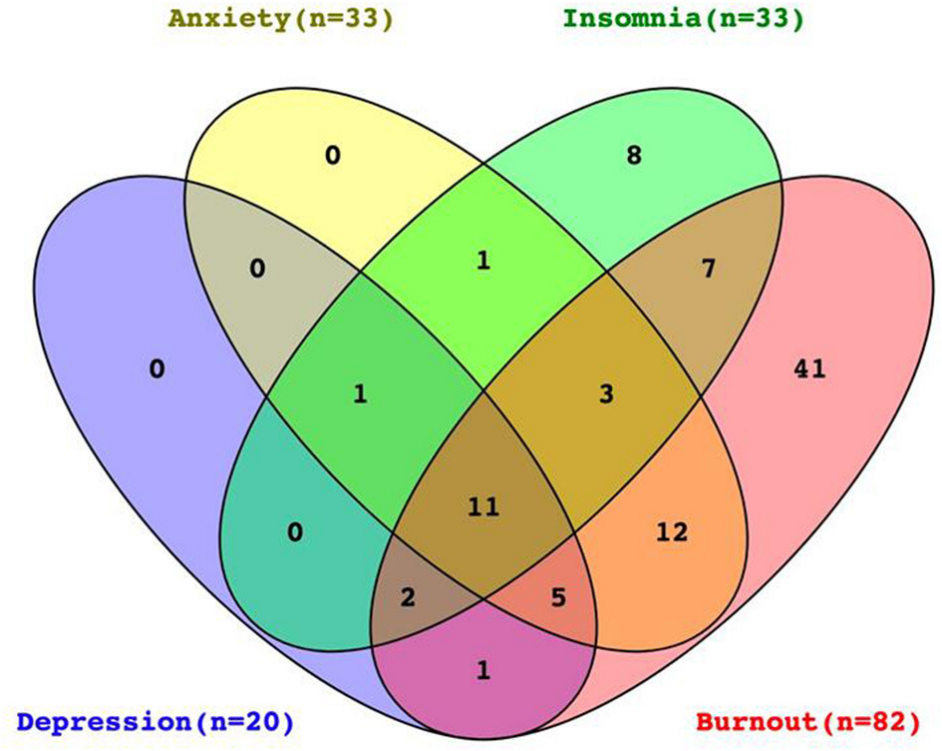
Venn diagram illustrates the degree of overlap between burnout status and clinically significant scores for depression, anxiety, and insomnia for participants with complete data on all four measures (*N* = 173). Ellipses of four colors, respectively, represent different adverse mental statuses. Yellow represents anxiety (*n* = 33), purple is depression (*n* = 20), green is insomnia (*n* = 33), and red is burnout (*n* = 82). The public collection part of ellipses means participants had two or more mental problems at the same time, and the figure in the ellipse represents the number of participants with this kind of psychological problem.

There was a significant degree of overlap between burnout status and clinically significant level of depression (*n* = 14), insomnia (*n* = 23), and anxiety (*n* = 31), most notably for anxiety (odds ratio, 27.049; 95% CI, 6.125–117.732; *p* < 0.001). A total of 11 participants were at risk of burnout, clinical depression, anxiety, and insomnia at the same time (Figure 1).

### Univariate analyses

Table 2 shows the association between burnout and socio-demographics of participants, adverse mental health status, coping style, and self-efficacy. The results indicate that participants with smoking present significantly reduced PA compared with no smoking. Participants reporting low PA were less likely to cope actively. Participants in reduced PA showed a significantly low level of self-efficacy. Low PA was not related to the job nature of healthcare workers and other occupational and socio-demographic factors (except for smoking). More than 90% of participants in the present study showed a high risk of reduced PA regardless of their emotional status and sleep quality. There was no significant association between depression, anxiety, or insomnia with reduced PA.

**Table 2:** Comparison in the associated factors of burnout and reduced PA (*N* = 173).

**Variable**		**Reduced PA (*n*, %)**	**Burnout (EE or DP) (*n*, %)**
Age group (year)	< 30	39, 92.86	20, 47.62
	30–39	74, 93.67	40, 50.63
	≥40	47, 90.38	22, 42.31
χ^2^		0.498	0.873
*p*		0.779	0.646
Gender	Male	66, 89.19	36, 48.65
	Female	94, 94.95	46, 46.46
χ^2^		2.022	0.081
*p*		0.155	0.776
Marital status	Others	53, 92.98	29, 50.88
	Married	107, 92.24	53, 45.69
χ^2^		0.030	0.413
*p*		0.862	0.521
Education	High school and below	5, 100.00	2, 40.00
	College	131, 91.61	67, 46.85
	Postgraduate degree	24, 96.00	13, 52.00
χ^2^		1.009	0.339
*p*		0.604	0.844
Household income	Low	41, 95.35	26, 60.47
	General	107, 92.24	53, 45.69
	High	12, 85.71	3, 21.43
χ^2^		1.441	6.868
*p*		0.487	0.032^*^
Profession	Medical care personnel	54, 93.10	23, 39.66
	Public health personnel	95, 92.23	55, 53.40
	Others	11, 91.67	4, 33.33
χ^2^		0.053	3.834
*p*		0.974	0.147
Years since qualified	< 5	44, 93.62	24, 51.06
	5–10	32, 96.97	15, 45.45
	>10	84, 90.32	43, 46.24
χ^2^		1.667	0.354
*p*		0.434	0.838
Work nature	Epidemiological survey	50, 90.91	34, 61.82
	Sampling	46, 92.00	20, 40.00
	Others	64, 94.12	28, 41.18
χ^2^		0.474	6.740
*p*		0.789	0.034^*^
Years of controlling COVID-19	< 0.5	39, 95.12	21, 51.22
	0.5–1	28, 93.33	16, 53.33
	>1	93, 91.17	45, 44.12
χ^2^		0.693	1.104
*p*		0.707	0.576
Physical condition	Well	73, 92.41	28, 35.44
	General	79, 94.05	48, 57.14
	With illness	8, 80.00	6, 60.00
χ^2^		2.539	8.365
*p*		0.281	0.015^*^
Smoking	No	14, 77.78	71, 45.81
	Yes	146, 94.19	11, 61.11
χ^2^		6.253	1.515
*p*		0.012^*^	0.218
Drinking	No	36, 90.00	58, 43.61
	Yes	124, 93.23	24, 60.00
χ^2^		0.463	3.314
*p*		0.496	0.069
Depression	No	142, 92.81	63, 41.18
	Yes	18, 90.00	19, 95.00
χ^2^		0.201	20.552
*p*		0.654	0.000^***^
Anxiety	No	129, 92.14	51, 36.43
	Yes	31, 93.94	31, 93.94
χ^2^		0.124	35.427
*p*		0.725	0.000^***^
Insomnia	No	130, 92.857	59, 42.14
	Yes	30, 90.91	23, 69.70
χ^2^		0.146	8.132
*p*		0.703	0.004^**^
Tendency of coping (*z*-value)		160, −0.089 ± 1.095	82, −0.421 ± 1.065
*t*		−3.780	−4.955
*p*		0.000^***^	0.000^***^
Level of self-efficacy		160, 2.32 ± 0.602	82, 2.28 ± 0.589
*t*		*-*3.390	−1.590
*p*		0.001^**^	0.114

* *p* < 0.05,

** *p* < 0.01, and

*** *p* < 0.001.

Participants with low income were more likely to be at high risk of burnout compared with the ones with high income. Participants working in the epidemiological survey showed a significantly high risk of burnout than others. Compared with participants with no illness, the ones with illness presented a significantly high risk of burnout. Participants reporting burnout were more likely to be depressive, insomniac, and coping negatively, compared with staff not at high risk of burnout. There was no significant difference in the level of self-efficacy between the group with burnout and the group without burnout. No significant findings were presented in the association of age, gender, marital status, educational level, profession, years since qualified, years of controlling COVID-19, drinking, and smoking with burnout.

### Hierarchical logistic regression

Those variables significantly associated with burnout in the univariate analysis entered the hierarchical logistic regression model for burnout. Socio-demographics and adverse mental states of depression, anxiety, and insomnia were added in model 1, and other variables significantly associated such as negative coping style were added in model 2. There was a significant additional improvement in fit for the models for burnout outcomes after the negative coping style was added, with increases in Nagelkerke Pseudo *R*^2^ of 0.052 for burnout. In model 1, the result showed that the work nature of the epidemiological investigation was associated with two times the risk of endorsing burnout. Anxiety was an independent risk factor for burnout. In model 2, the work nature of the epidemiological investigation was not still associated with the high risk of burnout, whereas anxiety was still associated with the high risk of burnout, and coping style was significantly negatively correlated with the high risk of burnout. Physical condition, depression, and insomnia were not independent risk factors of burnout. The results are presented in Table 3.

**Table 3:** Hierarchical logistic analysis of burnout and significant factors associated.

**Variables**	**Model 1**			**Model 2**		
	**OR (CI)**	**B**	***P***	**OR (CI)**	**B**	***P***
**Household income**						
Low	–	–	–	–	–	–
General	Reference	Reference	Reference	Reference	Reference	Reference
High	–	–	–	–	–	–
**Work nature**						
Epidemiological survey	2.380 (1.074–5.273)	0.867	0.033^*^	2.072 (0.905–4.743)	0.729	0.085
Sampling	0.664 (0.273–1.618)	−0.409	0.368	0.554 (0.218–1.403)	−0.591	0.213
Others	Reference	Reference	Reference	Reference	Reference	Reference
**Physical condition**						
Well	–	–	–	–	–	–
General	Reference	Reference	Reference	Reference	Reference	Reference
With illness	–	–	–	–	–	–
**Depression**						
Anxiety	32.050 (7.127–144.122)	20.434	0.001^**^	23.889 (5.216–109.414)	3.173	0.000^***^
**Insomnia**						
Negative coping	–	–	–	1.869 (1.261–2.770)	0.625	0.002^**^

* *p* < 0.05,

** *p* < 0.01, and

*** *p* < 0.001.

## Discussion

We studied 173 healthcare workers participating in our survey on the topic of burnout and associative emotional status and coping style. This study confirmed a lower prevalence of depression, anxiety, and insomnia among health professionals during the COVID-19 pandemic, compared with the reviews of the prevalence of mental status among healthcare workers by Mahmud et al. (27) and by Sahebi et al. (28) and also with the review of the prevalence of mental health problems among the global population (29). However, our findings of the prevalence rate of anxiety and insomnia were higher, and the rate of depression was lower than that reviewed by Xiong et al. (30). The significant level of heterogeneity of prevalence could be due to the stigma of psychological problems among medical staff, psychological measurement tools, cutoff value, and the phases and conditions of the COVID-19 pandemic in different studies. This study chose a high threshold value, and the physical and mental status of participants in this study could adapt and adjust more than 2.5 years after the COVID-19 outbreak, which may partly explain the lower prevalence rate of depression, anxiety, and insomnia. Whether the prevalence of psychological problems will be decreasing or not in future needs to be confirmed by other studies, and further research is needed in the area.

The findings presented that nearly half of the health professionals in our study had experienced a high risk of burnout. In addition, approximately a third, over 40%, and more than 90% of these workers showed severe levels of EE, DP, and low PA, respectively. These values of EE, DP, and PA were slightly different in the studies (31–34). Hyman et al. found a higher EE value and a lower DP and reduced PA in anesthesiologists than in our survey. A national cross-sectional study in China before the outbreak of COVID-19 showed that the high-risk rate of burnout was 44.2% (32). In one other study, 9.7% of Indian healthcare workers during the early stage of the COVID-19 pandemic were showing a high risk of burnout (35), which was lower than our result. The average age of subjects in our study was 35 years old; however, some research showed that being relatively young often tended to a high risk of burnout (34). The discrepancy in the prevalence rates of burnout in the given study could be associated with the COVID-19 pandemic time, place, and other conditions such as work factors. This study was carried out during the COVID-19 pandemic, and we think COVID-19 is an important influencing factor of burnout; however, the present research cannot completely rule out the influence of work factors on burnout, and further research is needed to clarify the relationship between work factors and burnout.

It was worth noting that our study showed a relatively high value in reduced PA than other studies (32, 35, 36). Not like the associated factors of burnout in this finding, the high level of reduced PA of healthcare workers for controlling the epidemic was not related to negative mental emotions of depression or anxiety and insomnia. The level of self-efficacy and coping style affected the PA value of participants. Subjects with reduced PA showed a low level of self-efficacy. Healthcare workers with a low sense of achievement were more likely to adopt negative coping such as smoking in the present study. PA was associated with job satisfaction. The high level of reduced PA in our study could be attributed to the fact that participants were involved in work of COVID-19 prevention and control for more than 2 years, rather than the daily work that they are good at. Otherwise, these professionals worked in centralized isolated conditions, isolated from society and family, in a hall where the sun did not shine and they could not tell day from night. Furthermore, they had to sacrifice personal time and spend more time on work and needed to participate in epidemic response as needed at any time. Medical staff in epidemic control had to waste time on complex paperwork required for reporting and meetings. Inevitably, little work value and too much energy consumption among participants in this study would lead to low personal accomplishment.

A significant relationship had been observed between anxiety, depression, insomnia, passive coping, and burnout in our study. This study showed a high overlap between anxiety and burnout. The results presented that participants with a high risk of burnout were highly comorbid with anxiety especially, and anxiety was a significant independent high-risk factor of burnout. Emotional illness was one of the key obstacles for medical staff (37). The study by Reitz et al. revealed reducing the detrimental effect of anxiety may reduce the risk of burnout among healthcare providers (38). In one other study, Sun et al. tested that anxiety and depression as a potential moderating effect worsened occupational burnout (39). Furthermore, Deneva et al. verified a significant positive correlation between burnout and depression (40). In contrast to the reports earlier, in the present study, depression was not significantly independent relative to burnout. The contradictory results need further research to explore the relationship between burnout and depression. However, the consensus in previous and present studies was that there was a clear correlation between anxiety and burnout. Ameliorative strategies to reduce anxiety could be used to mitigate burnout among healthcare workers. Not like other studies, the results in the present study did not show any significantly associated socio-demographic factors such as age, gender, marital status, or occupation of burnout.

Coping passively was an independent risk associated with burnout observed in the present study. Liu et al. and Yu et al. similarly reported that burnout was positively associated with passive coping styles among Chinese nurses (41, 42). Coping style plays a mediating role in burnout (43), for example, negative coping styles mediate the association between burnout and anxiety symptoms in Chinese physicians (44). Although some surveys explored the relationship between burnout and coping or adverse mental status, less was available about how the level of self-efficacy affected burnout among healthcare workers. The high risk of burnout in vascular surgery trainees was associated with higher levels of depression and lower levels of self-efficacy reported by Janko et al. (45). Yao et al. (46) showed that self-efficacy as an important and protective factor did a mediating effect of stress on job-related burnout. In contrast, this study explored the relative impact of self-efficacy on the outcome of burnout, and no significant relationship had been observed between self-efficacy and burnout.

There are some innovations in this study. First, the survey was carried out in a centralized isolated environment and a special work mode of long-term separation of participants from family. Those subjects faced high stress and uncertainty. Second, many studies had noticed anxiety, depression, and insomnia in medical staff, and we further explored the association between burnout and adverse emotional status, insomnia, coping style, and level of self-efficacy. The results showed that the incidence of burnout was high, especially the low PA, which is rare in other studies. Finally, this study is realistic and has a certain reference value for similar research work in future.

In the past 2 years and more during the COVID-19 pandemic, it was difficult for most humans, especially the medical staff to manage epidemic prevention. No one could tell them when the epidemic would end and when they could return to their original jobs. China faces and will face multiple peaks of COVID-19 infection like the rest of the world. Healthcare workers are committed to guarding the life and health of the public, and it needs to pay attention to their mental health, dilute medical exception theory, and ease emphasis on personal responsibility. It is important to assess the mental status and adopt multiple coping strategies and improve self-efficacy to deal with burnout during and after the COVID-19 pandemic.

Care and support for medical staff should be strengthened. Health authorities and institutional leaders should take the responsibility for promoting the health of the medical workplace and improving the health of the medical staff from the system level. The psychological health problems of the staff need to be paid attention to. Medical administrators need actively understand and coordinate to solve the actual difficulties and needs of the medical staff and take a systematic approach to solve the burnout and mental problems of the medical staff, such as eliminating the stigma of psychological problems, providing psychological resources, actively creating a good working environment, limiting working hours, reducing workload, and providing individually tailored mental health protection.

## Limitations

Although there are important discoveries revealed by this study, there are also limitations. First, this is a cross-sectional survey with a small sample size, which limits the generalizability of the results. Second, our results can only present an association rather than causation between anxiety, coping style, and burnout. Third, it is hard to fully rule out reporting errors by self-reported data, and subjects may be affected by recall bias and high social expectations for medical staff, who bear more personal responsibilities, medical missions, or commitments, face the stigma of mental health problems, and find it more difficult to actively express their psychological discomfort. Finally, although younger people were more affected by major depressive disorder and anxiety disorders, our results may not be generalized to all professionals as the study sample was relatively young (mean = 35 years) and might face greater social and occupational stress and burnout in China.

## Conclusion

This study confirmed the previous findings that burnout was very common among medical staff. Participants involved in COVID-19 epidemic control in the post-epidemic era were at high risk of burnout, and most of them were in low personal accomplishment. Anxiety and negative coping styles were significantly and independently associated risk factors for burnout among healthcare workers. Reducing anxiety and improving coping style may effectively alleviate burnout in healthcare workers. Medical management institutions should provide care and support from the system level for medical staff.

## Data availability statement

The raw data supporting the conclusions of this article will be made available by the authors, without undue reservation.

## Ethics statement

The studies involving human participants were reviewed and approved by Longgang Center for Chronic Disease Control, Shenzhen. The patients/participants provided their written informed consent to participate in this study.

## Author contributions

CY and CW conceived and designed the experiments. CY and XW performed the experiments. XZ and WL analyzed the data. CY, XW, and CW wrote the manuscript. All authors contributed to the article and approved the submitted version.
